# Supplementary material for: Building a queer- and trans-inclusive microbiology conference
Source: mSystems. 2023 Oct 6;8(5):e00433-23. doi: 10.1128/msystems.00433-23 (PMC10783533; doi:10.1128/msystems.00433-23)
Supplement: Supporting Information — Author positionality statement and additional resources. [file msystems.00433-23-s0001.docx]

**Supporting Information**

**Building a Queer- and Trans-Inclusive Microbiology Conference**

Rachel Gregor,^1^*^ Julie Johnston,^2^^ Lisa Shu Yang Coe,^3#^ Natalya Evans,^4#^ Desiree Forsythe,^#5^ Robert Jones,^6#^ Daniel Muratore,^7#^ Bruno Francesco Rodrigues de Oliveira,^8#^ Rachel Szabo,^1#^ Yu Wan,^9#^ Jelani Williams,^#10^ Callie R. Chappell,^11$^ Shayle B. Matsuda,^12$^ Melanie Ortiz Alvarez de la Campa,^13$^ Queer and Trans in Microbiology Consortium, JL Weissman,^5,10^*^

1. Massachusetts Institute of Technology, Department of Civil and Environmental Engineering, Cambridge, MA, USA
2. Georgia Institute of Technology, School of Civil and Environmental Engineering, Atlanta, GA, USA
3. New York University Abu Dhabi, Division of Science, Biology Program, Abu Dhabi, UAE
4. University of California, Santa Barbara, Santa Barbara, CA, USA
5. Schmid College of Science and Technology, Chapman University, Orange, CA, USA
6. U.S. Army Engineer Research and Development Center, Cold Regions Research and Engineering Laboratory, Hanover, NH, USA
7. Santa Fe Institute, Santa Fe, NM, USA
8. Fluminense Federal University, Department of Microbiology and Parasitology, Biomedical Institute Niterói, RJ, Brazil
9. Imperial College London, Faculty of Medicine, Department of Infectious Disease, London, UK
10. University of Southern California, Department of Biological Sciences, Los Angeles, CA, USA
11. Stanford University, Department of Biology, Stanford, CA, USA
12. Daniel P. Haerther Center for Conservation and Research, John G. Shedd Aquarium, Chicago, IL, USA
13. Brown University, Department of Molecular Microbiology & Immunology, Providence, RI, USA

*Corresponding: rgregor@mit.edu (RG), jw4336@terpmail.umd.edu (JLW)

^Authors contributed equally. Author order was determined both by additional roles as corresponding authors and by increasing seniority (due to differing preferences for first/last position based on career stage)

^#^Authors contributed equally and are listed alphabetically

^$^Authors contributed equally and are listed alphabetically

**S1 Text: Positionality statement:**

This paper is based on the personal experiences of the authors. Our author team encompasses a broad range of lived experiences and queer and trans identities, but does not claim to be comprehensive or representative of all queer and trans microbiologists. Additional perspectives, insights, and feedback were contributed by Queer and Trans in Microbiology Consortium members. The identities below were polled using a free response form, allowing authors to highlight any aspects of their identities they wished to.

All authors fall under the umbrella of LGBTQ+, with gender identities including nonbinary, genderqueer, genderfluid, woman of transgender experience, trans woman, man of transgender experience, cis male, and cis woman; and sexualities including queer, bisexual, pansexual, lesbian, homosexual, and gay. Of the 15 listed authors, 10 identify as trans, non-binary, genderqueer, and/or genderfluid. Our author list also includes identities such as Afro-Brazilian, Afro-Caribbean, Asian American, Black, Chinese, Cuban, Hispanic, Japanese, Jewish, Puerto-Rican, white, neurodivergent, disabled, trans-racial adoptee, able-bodied, and first-generation. The authors are predominantly United States of America nationals, with additional nationalities including Brazilian, Chinese, Israeli, Jamaican, and Puerto Rican.

The authors are all early-career, including researchers, post-doctoral researchers, graduate students, laboratory technicians, and early career faculty, based in primarily academic institutions as well as government, predominantly in the United States of America as well as the United Arab Emirates, Brazil, and the United Kingdom.

**S2 Text: Further resources and guides**

**General best practices for inclusion**

- [Supporting LGBTQ+ geoscientists, in and out of the classroom by Downen and Olcott (2022)](https://www.tandfonline.com/doi/full/10.1080/10899995.2022.2116205) (https://www.tandfonline.com/doi/full/10.1080/10899995.2022.2116205)
- [Supporting LGBTQ students by OSTEM](https://ostemnorthtexas.github.io/site/pages/LGBTQ-students/) (https://ostemnorthtexas.github.io/site/pages/LGBTQ-students/)
- [LGBT+ Inclusivity in Physics and Astronomy - A best practices guide by Ackerman et al., 2018](https://arxiv.org/abs/1804.08406) (https://arxiv.org/abs/1804.08406)

**Trans-specific practices for inclusion**

- [Creating a Trans-Inclusive Workspace by Thoroughgood et al., 2020](https://hbr.org/2020/03/creating-a-trans-inclusive-workplace) (https://hbr.org/2020/03/creating-a-trans-inclusive-workplace)
- [Transgender Ally by the LGBTQ+ Student Center](https://lgbtqplus.usc.edu/services-and-resources/transusc/transgender-ally/) USC (https://lgbtqplus.usc.edu/services-and-resources/transusc/transgender-ally/)
- [Action Tips for Allies of Trans People by MIT](https://customsitesmedia.usc.edu/wp-content/uploads/sites/373/2015/05/18000245/Actions-Tips-for-Allies-of-Trans-People1.pdf)

(https://customsitesmedia.usc.edu/wp-content/uploads/sites/373/2015/05/18000245/Actions-Tips-for-Allies-of-Trans-People1.pdf)

**Language and pronouns guides**

- [LGBTQUIA+ Inclusive Language](https://warwick.ac.uk/fac/cross_fac/academy/activities/learningcircles/transqueerpedagogies/queeringuniversity/resources/inclusivelanguage/) (https://warwick.ac.uk/fac/cross_fac/academy/activities/learningcircles/transqueerpedagogies/queeringuniversity/resources/inclusivelanguage/)
- [A beginner’s guide to pronoun usage](https://www.stonewall.org.uk/workplace-trans-inclusion-hub/beginner%E2%80%99s-guide-pronouns-and-using-pronouns-workplace) in the workplace (https://www.stonewall.org.uk/workplace-trans-inclusion-hub/beginner%E2%80%99s-guide-pronouns-and-using-pronouns-workplace)
- [Pronouns: A Guide for the American University Community](https://www.american.edu/ocl/cdi/pronouns-guide.cfm) (https://www.american.edu/student-affairs/cdi/pronouns-guide.cfm)

**Inclusion practices for scientists with disabilities, chronic illness, and/or neurodiversity**

- [Nothing About Us Without Us – Towards Genuine Inclusion of Disabled Scientists and Science Students Post Pandemic (2021)](https://www.ncbi.nlm.nih.gov/pmc/articles/PMC8361768/) (https://www.ncbi.nlm.nih.gov/pmc/articles/PMC8361768/)
- [How to Make Professional Conferences More Accessible for Disabled People: Guidance from Actual Disabled Scientists (2018)](https://blog.ucsusa.org/science-blogger/how-to-make-professional-conferences-more-accessible-for-disabled-people-guidance-from-actual-disabled-scientists/) (https://blog.ucsusa.org/science-blogger/how-to-make-professional-conferences-more-accessible-for-disabled-people-guidance-from-actual-disabled-scientists/)

**Additional guides for fieldwork and marine research**

- [Gender inclusion at sea](https://agupubs.onlinelibrary.wiley.com/doi/full/10.1029/2023AV000927): https://agupubs.onlinelibrary.wiley.com/doi/full/10.1029/2023AV000927
- [Best practices for LGBTQ+ inclusion during ecological fieldwork: Considering safety, cis/heteronormativity and structural barriers](https://doi.org/10.1111/1365-2664.14339) (https://besjournals.onlinelibrary.wiley.com/doi/10.1111/1365-2664.14339)

**S3 Text: Example of information to provide to LGBTQ+ attendees before a conference.**

*Note: The below text appeared on the website for the 2022 Conference on Empirical Methods in Natural Language Processing, which took place in Abu Dhabi, UAE. It has been reproduced here with the author’s permission, and was originally available here: https://2022.emnlp.org/blog/EMNLP-2022-Abu-Dhabi-LGBTQ+-Visitor-Considerations/*

**LGBTQ+ Visitor Considerations**

This blog post provides some insights and advice for LGBTQ+ visitors by LGBTQ+ people living in Abu Dhabi.

**Author and Audience**

The primary author of this document is a cisgender gay Arab-American man. He has lived in the UAE with his cisgender gay European-American partner for almost a decade. They both have academic jobs, and love living in the UAE.

The author’s advice and observations are based on his experience of living in the UAE, and his awareness of issues faced by other members of the LGBTQ+ community there. The intended audience of this document are LGBTQ+ conference attendees of EMNLP 2022.

This document is not intended to provide official legal advice.

**Many thanks to all the community members (LGBTQ+ and allies) who helped with reviewing and editing different versions of this document.**

**The Public and The Private**

Emirati culture values a separation between public and private lives in a way that’s different from some Western nations. In Abu Dhabi, regardless of gender identity or sexual orientation, public displays of affection are generally frowned upon, especially prolonged intimate actions. There is no dress code but general modesty for both men and women is the expected norm. **Beyond that, privacy is highly respected: what one does in their own home is their business. In in-between public-private spaces, such as hotels, the expectation for all unmarried couples (gay or straight) is to have separate beds**. These can be two double beds. But no one will check what happens in the room.

**Diversity and Tasamuh**

The UAE is very diverse in terms of the countries of origin of its residents – with close to 90% non-Emiratis and 30% non-Muslims. The diverse backgrounds have created a regional norm around different cultures coexisting. The UAE’s attitude about the diversity of its residents is that of “Mutual Tolerance,” the Arabic concept of تسامح tasāmuh, which comes from the root smh associated with concepts like forgiveness, permission, tolerance, magnanimity, and the pattern ta-ā-u- of mutuality. In other words — **live and let live**. The UAE even has a dedicated [Ministry of Tolerance](https://www.google.com/url?q=http://www.tolerance.gov.ae/en/default.aspx&sa=D&source=docs&ust=1664870428955606&usg=AOvVaw1QfS0UTuPT0S7zjBzCCB_G).

To date, this concept has not been explicitly expanded to include sexual orientation or gender identity. However, in practice, accepting cultural diversity indirectly includes accepting some sexual and gender diversity since mutual tolerance covers opinions held by people from different cultural backgrounds. Also, perhaps more subtly, the cultural diversity in the UAE leads to more tolerance of diversity of gender expression; **what some in the West would consider gay or queer may be a straight or unmarked behavior in other cultures. This goes beyond gender being a social construct, to it being constructed differently in different cultures**.

Examples abound in the UAE of behaviors that may be marked differently in different cultures:

- Men holding hands is not marked as gay among Arabs, but rather as indicator of close friendship.
- Men kissing men on the cheeks (typically 3-4 times alternating) is not marked at all and in some cases it is rude not to kiss back.
- Very subtle and specific behaviors that are caricatured gay stereotypes in the West like lifting the pinky when drinking from a cup is not marked in the Arab World at all. You will see men doing this; and it means nothing.
- Same-gender people living together as roommates is not marked as gay/lesbian. The default assumption is simply that they have families elsewhere and need to save money… or no explanation is needed.
- Emirati men sometimes greet each other with a so-called “[nose-kiss](https://www.youtube.com/watch?v=0_Nai5VZR8s)” that looks too intimate for non-Emiratis.
- While Emirati men typically wear white Kanduras (dresses), some Arab and also European men like to dress in a more colorful style than most American straight men do. The variability is not judged or marked in any way that connects it to gender/sexuality. Think of the Legally Blond song “[Gay or European](https://www.youtube.com/watch?v=XY9PmBNb3PE&t=85s)”. This ambiguity covers a wide range of androgynous hair and dress styles that are not marked.

**In sum, many of the public expressions of gender that visitors may embody are not consistently marked across all cultures; and with a multicultural society like the UAE, the signal is simply lost in the “noise of multiculturalism”**. So, for most cis/cis-passing folks, there is no need to stress about being perceived one way or another. Of course, the situation is more complex for transgender people. More on that below.

**Laws and Practices**

There are no federal LGBTQ+-specific laws in the UAE. However, some prohibitions intended to protect women can be interpreted as restricting gender expression. For example, UAE Penal Code Article 412 part 2 states that “any male who disguises as a woman, or enters a place allocated for women only, or to which entry of men is forbidden,” the penalties can include a fine of no more than AED10,000 or imprisonment of one year or both” ([More details here](https://www.google.com/url?q=https://lovin.co/dubai/en/latest/cross-dressing-is-criminalised-once-again-in-the-uae&sa=D&source=docs&ust=1664870677664940&usg=AOvVaw3WSjNEz0my6Zp9VzLrWpP8)). Several emirates, including Abu Dhabi and Dubai, have additional prohibitions on behaviors, but these laws are almost never invoked because of the country’s general respect of privacy for its residents. See [Human Rights Watch](http://internap.hrw.org/features/features/lgbt_laws/index-june15.html) for more details. Of course, this situation is not different from many places around the [world that still have archaic laws](https://en.wikipedia.org/wiki/Sodomy_laws_in_the_United_States#:~:text=Eleven%20states%27%20statutes%20purport%20to,Carolina%2C%20Oklahoma%20and%20South%20Carolina).

Up to January of 2022, UAE law prohibited cohabitation of unmarried straight couples; this since has changed. Homosexual couples never suffered this issue because they are seen as simply roommates.

**Trans and Non-binary Individuals**

Gender dysphoria is recognized as a medical condition in the UAE, and [gender affirming surgery is legal in the UAE](https://www.thenationalnews.com/uae/health/new-uae-law-allows-gender-reassignment-surgery-1.218691). The main challenge for transgender individuals is legal paper work (passport, id) that is not updated from their birth sex to match their current gender expression. In such cases, the individual may be denied entry if they present themselves in a way that mismatches their paperwork upon entry to the country.

The UAE, like many other countries, uses visual inspection of documents as the primary means of identification of travelers. This is unlike countries such as the United States, where biometrics (e.g., your fingerprints) are used. Visitors should make sure that pictures on any of their travel documents match their current appearance; if not, the visitor might be subject to additional scrutiny when going through passport control. The worst of it will be wasted time at the airport, and denial of entry. Examples of famous cases where this happened include the case of transgender models [Gigi Gorgeous](https://vidooly.com/blog/gigi-gorgeous-dubai-airport-detained/) and [Rachaya Noppakaroon](https://sea.mashable.com/life/19811/thai-trans-model-turned-away-at-dubai-airport-for-possessing-male-passport).

Some countries now issue non-binary gender classification (e.g. M/F/X). This is not an issue as UAE immigration follows international agreements between the UAE government and these countries’ governments to respect their documents. However, many countries still do not allow updating the sex on the passport.

**Unfortunately, for transgender individuals who do not have updated legal documents, our advice is to avoid the hassle and only participate virtually in the conference. This is grossly unfair of course. We hope these regulations will be changed in the future**.

**Online Sources of Mis/Information**

There is a lot of misinformation online about the UAE and LGBTQ+ issues. Some of the Wikipedia and Wikitravel pages are filled with inaccurate information on this topic. For example, the [wikipedia page on LGBTQ rights in the UAE](https://en.wikipedia.org/wiki/LGBT_rights_in_the_United_Arab_Emirates) claims that gay men are given the death penalty in the UAE; this is not true - the law is mistranslated from the Arabic (where it is specifically about rape). For some time, the [wikitravel](https://wikitravel.org/en/United_Arab_Emirates) page on the UAE claimed that there are “vigilante executions” against gay people in the UAE all the time with police complicity, which is outrageously false.

The following are reasonable articles reflecting the real situation from actual LGBTQ individuals who live or have visited the UAE:

- <https://nomadicboys.com/gay-life-in-dubai/>
- <https://nomadicboys.com/is-abu-dhabi-safe-for-gay-travellers/>
- <https://nomadicboys.com/gay-united-arab-emirates-is-dubai-safe-for-gay-travellers/>
- <https://queerintheworld.com/gay-abu-dhabi-uae-travel-guide/>

The following sites include helpful advice for all visitors:

- <https://www.worldnomads.com/travel-safety/middle-east/united-arab-emirates/what-you-can-or-cant-do-in-the-united-arab-emirates>
- <https://www.expatwoman.com/dubai/money-finance/finance/what-can-get-you-arrested-dubai-airports>

**Safety in a Broader Sense**

**Abu Dhabi is a very safe city, with a very low level of crime**. It compares very positively to other locations where other CL conferences were held this year as shown in the the following figure:


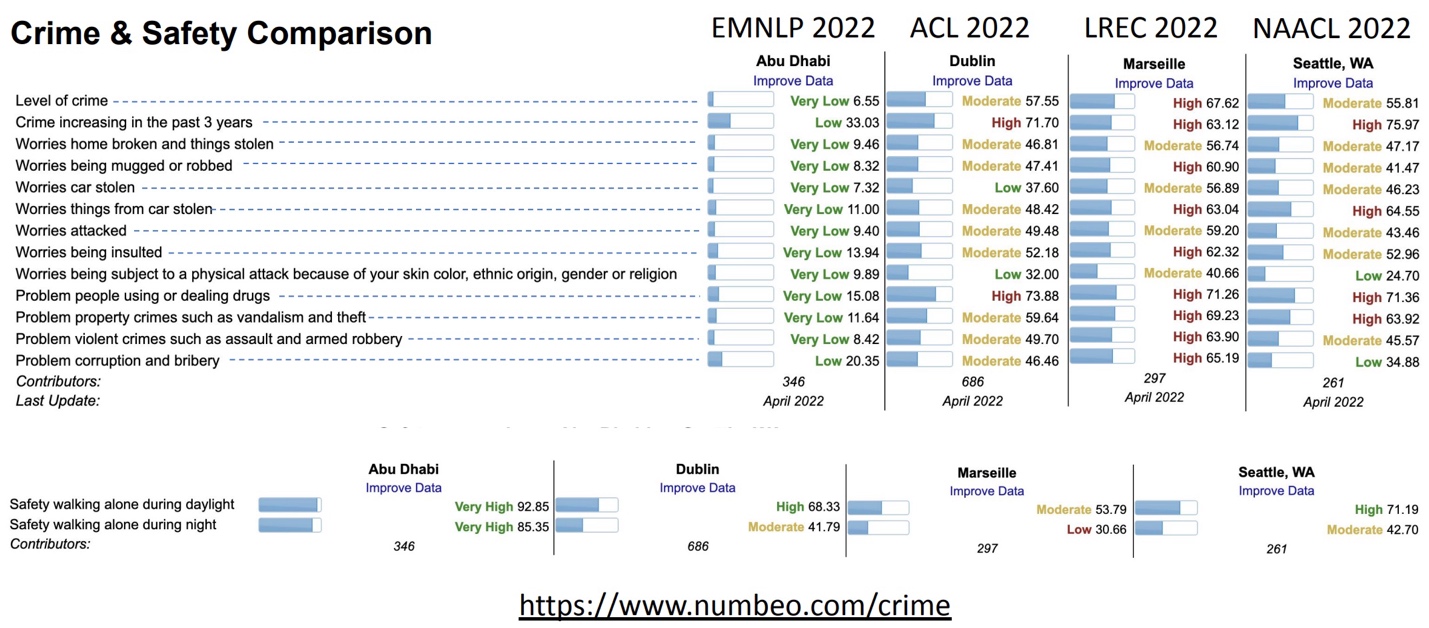


Furthermore, there are numerous international institutions that have branches in Abu Dhabi: [New York University](https://nyuad.nyu.edu/en/), [The Sorbonne University](https://www.sorbonne.ae/), [The Louvre Museum](https://www.louvreabudhabi.ae/), and others, many of whom employ and host LGBTQ people. [Expo 2020](https://www.expo2020dubai.com/) was a huge international event hosted in Dubai, with millions of visitors. All these people and organizations would not have chosen to be in the UAE if it was unsafe.

**Changing World**

The Arab World is not a monolith; it is a vibrant continuously evolving place. It may be behind the West in terms of LGBTQ liberation. But there are ongoing conversations and efforts in the public sphere in the Arab World about this issue. It just needs time. Not all countries will be on board and not at the same time. The UAE has impressively broken many political and social taboos that no one thought could be challenged in the last ten years: from peace with Israel to allowing unmarried cohabitation and open restaurants during the month of fasting, Ramadan.

On the LGBTQ liberation front, here are some links of interest:

- The Emirati Film (Only Men Go to the Grave)
  - <https://www.nbcnews.com/feature/nbc-out/emirati-filmmaker-pushes-boundaries-exposes-hidden-lives-n796351>
- The Netflix movie “Perfect Strangers” (Arab Version, partial funding from UAE)
  - <https://www.npr.org/2022/02/03/1077379012/netflix-egypt-perfect-strangers-mona-zaki>
  - <https://en.wikipedia.org/wiki/Perfect_Strangers_(2022_film)>
- Arab Drag Artist Bassem Feghali was featured in Vogue Arabia in the issue of September 2022. Vogue Arabia’s headquarter is in Dubai, UAE.
  - <https://en.vogue.me/fashion/lebanese-comedian-bassem-feghali-balenciaga-fall-2022-couture-video-pictures/>
  - <https://www.milleworld.com/meet-the-arab-worlds-drag-queens/>
- The HRW report: Audacity in Adversity: LGBT Activism in the Middle East and North Africa
  - <https://www.hrw.org/report/2018/04/16/audacity-adversity/lgbt-activism-middle-east-and-north-africa>
- Of course all of this is not happening without some resistance:
  - <https://www.cnbc.com/2022/09/07/saudi-arabia-and-gulf-neighbors-threaten-netflix-over-content-that-violates-islamic-values.html>
  - <https://www.khaleejtimes.com/education/uae-ministry-adopts-code-of-conduct-for-education-sector>

Conferences and other international events like EMNLP actively participate in this change - through a combination of expressed reservations (thanks to all those who tweeted their concerns!) as well as presenting role models of successful LGBTQ people to new communities (advanced thanks to all those who will join us in Abu Dhabi in person or virtually).

**Tags:**[announcement](https://2022.emnlp.org/tags/#announcement) [publicity](https://2022.emnlp.org/tags/#publicity)

**Categories:**[blog](https://2022.emnlp.org/categories/#blog)

**Updated:** October 04, 2022
